# Obesity as a risk factor for cardiac arrhythmias

**DOI:** 10.1136/bmjmed-2022-000308

**Published:** 2022-10-19

**Authors:** Kiran Haresh Kumar Patel, Rohin K Reddy, Arunashis Sau, Pavidra Sivanandarajah, Maddalena Ardissino, Fu Siong Ng

**Affiliations:** 1 National Heart and Lung Institute, Imperial College London, London, UK; 2 Nuffield Department of Population Health, University of Oxford, Oxford, UK

**Keywords:** cardiology, electrophysiology, metabolic diseases

## Abstract

Obesity is global health problem with an estimated three billion people worldwide being classified as overweight or obese. In addition to being associated with a range of adverse health outcomes, obesity is linked to higher risks of atrial and ventricular arrhythmias, as well as sudden cardiac death. Obesity is a multifactorial disease that often co-exists with hypertension, diabetes, and sleep apnoea, which are also independent risk factors for cardiac arrhythmias. Nevertheless, compelling evidence suggests that increasing adiposity is an independent proarrhythmic risk factor and that weight loss can be a mitigating and preventative intervention to reduce arrhythmia incidence. This review briefly outlines the economic and social burden of obesity and summarises evidence for the direct and indirect effects of increasing adiposity on risk of atrial and ventricular arrhythmias. The paper also summarises the evidence for electrocardiographic changes indicative of obesity-related atrial and ventricular remodelling and how weight reduction and management of comorbidity might reduce arrhythmic burden.

## Introduction

Approximately 3 billion people, or half the world’s population, is either overweight or obese.^1^ The worldwide prevalence of obesity has tripled since 1975, and is thus an increasingly important global economic and health burden.^2^ Body mass index is the ratio of weight to height and forms the basis for most healthcare recommendations and guidelines because it is quick and easy to calculate, enabling tracking of changes in whole body adiposity over time. A healthy body mass index is considered 18.5-24.9, overweight is 25.0-29.9, and obesity is 30.0 or more. Subclassifications include grade 1 obesity, defined as 30.0-34.9, grade 2 as 35.0-39.9, and grade 3 as 40.0 or more. However, these definitions are flawed because body mass index is an imprecise measure of adiposity. UK obesity management guidelines^3^ suggest that waist girth, an indicator of abdominal visceral adiposity, might therefore be a useful adjunct to measure health risks in individuals who are overweight or obese. Other anthropometric indices can quantify adiposity, such as waist to hip ratio and waist to height ratio, although these indices do not yield substantially more information on adiposity than can be gained from waist girth. Abdominal diameter is a surrogate for waist girth but is more difficult to measure clinically.^4^ For these reasons, body mass index and waist girth are the mainstay metrics for quantifying weight and adiposity in guidelines.^3 5 6^ In this review, we summarise evidence for obesity as a risk factor for atrial and ventricular arrhythmias, and the extent to which these are conferred by increasing adiposity or mediated by cardiometabolic comorbidity. We also explore evidence for the effects of lifestyle and surgical weight management, and management of comorbidity related to obesity that might mitigate arrhythmic risks.

## Epidemiology of obesity and related arrhythmias

Obesity affects more than 600 million adults worldwide.^7^ The increasing prevalence of obesity in low and middle income countries is attributed to urbanisation, easy accessibility to processed energy-dense food, and cultural shifts towards lifestyles of high income countries.^8^ Two thirds of the UK population is overweight or obese.^9^ In 2011, modelling suggested that by 2030 the number of obese adults would increase by 65 million in the US and by 11 million in the UK. As a consequence, the number of patients in the UK with diabetes would increase by up to 8 million, for heart disease and stroke by 7 million, and for cancer by 0.5 million, at the expense of 55 million quality adjusted life years, adding £2000 million (€2.3 billion; $2.2 billion) to healthcare costs each year.^10^ Another meta-analysis estimated that, compared with individuals of healthy weight, UK healthcare costs increase by as much as 12% for excess weight and 36% for obesity, secondary to greater demand for drug treatments, hospital admission, and ambulatory care.^11^ Indeed, prescription costs incurred by a patient who is morbidly obese over a three year period are 10 times greater than that of someone of a healthy weight.^12^ Given that ageing confers a higher risk of arrhythmias and the increase in ageing populations in high income countries,^13^ evidence linking increasing adiposity and atrial and ventricular arrhythmias presented in this review suggests that the obesity epidemic is likely to add to the burden of arrhythmias in the future.

## Sources and selection criteria

We searched PubMed and Embase databases for clinical studies in the English language that were published between 1 January 1980 and 1 April 2022. Studies must have evaluated arrhythmic risks and electrocardiographic adaptations associated with obesity, or cardiovascular outcomes associated with weight reduction strategies in obesity. We used the following search terms: "obesity," "arrhythmia," "atrial arrhythmia," "atrial fibrillation OR AF," "ventricular arrhythmia," "QT OR QTc," "P wave," "electrocardiographic," "OSA OR sleep apnoea OR sleep apnea OR obstructive sleep apnoea," "diabetes," "hypertension," "weight loss OR weight reduction," "bariatric surgery." We prioritised peer reviewed randomised controlled trials and cohort studies and excluded case studies or series. In addition to primary sources, we searched reference lists as additional sources of information.

## Obesity related atrial remodelling

Atrial dilatation and consequent electroanatomic architectural distortion causes conduction slowing and increases conduction heterogeneity, which are hallmarks of a proarrhythmic substrate.^14^ In multivariable analyses of 13 382 echocardiograms, which were adjusted for age and sex, patients who were obese had increased odds of left atrial enlargement (odds ratio 2.53 (95% confidence interval 2.30 to 2.75)), in addition to other parameters of diastolic dysfunction.^15^ A prospective study of 1212 adults who were followed up over 10 years showed that obesity and hypertension were independent predictors of left atrial volume after adjusting for age, sex, cardiovascular disease, and other risk factors.^16^ Furthermore, linear regression analyses showed that the effect of obesity was almost twice that of hypertension. The Framingham Heart Study (FHS), a prospective, community based observational study of 5282 participants, also showed that left atrial dilatation was a mediator of atrial arrhythmias associated with increasing body mass index.^17^

Atrial electroanatomic adaptations related to obesity manifest as changes in P wave indices on 12 lead electrocardiogram, including P wave duration and dispersion, which characterise right and left atrial electrical depolarisation.^18^ P wave indices have been associated with atrial remodelling in an echocardiographic study,^19^ as well as with increased risk of atrial fibrillation (AF) in the Atherosclerosis Risk in Communities study^20^ and in the FHS.^21 22^ Furthermore, investigation of 14 433 participants in the Atherosclerosis Risk in Communities study showed progressive abnormalities in the P wave indices with increasing weight after adjustment for cardiovascular risk factors.^23^ Correspondingly, reverse atrial remodelling denoted by regression of adverse P wave indices correlates with degree of weight loss in obesity.^24^

## Obesity as a risk factor for atrial fibrillation

**Figure 1:**
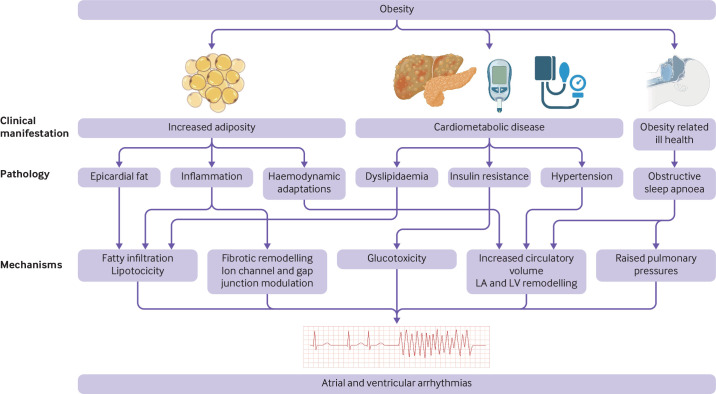
Mechanisms underpinning obesity as a risk factor for atrial and ventricular arrhythmias. The higher risk of cardiac arrhythmias in obesity is conferred by increasing adiposity, concurrent cardiometabolic disease, and related ill health. These result in adverse electrophysiological remodelling of the atria and ventricles to create a proarrhythmic substrate. The evidence for some of these mechanisms and their potential for reversibility with weight reduction are discussed in the main text. LA=left atrium; LV=left ventricle

AF is the most common sustained arrhythmia.^25^ Strong clinical evidence from prospective and retrospective studies links obesity to a higher AF incidence.^26 27^ A meta-analysis of 51 studies including 626 603 participants showed that a 5 unit increase in body mass index conferred 29% higher odds of incident AF in cohort studies and 19% higher odds in case control studies.^26^ Odds of postoperative AF resulted in 10% greater per 5 unit increase in body mass index, as was the case after ablation AF, which reported a 13% greater risk, suggesting that AF recurrence after invasive treatment is greater in people with higher body mass.^26^ Additionally, a nationwide cohort study showed that although AF incidence was low among more than 270 000 young women (mean age 31 years, standard deviation 5 years), obesity still independently predicted AF in this age group.^28^ Furthermore, in a prospective, community cohort study that followed up 3000 patients for six years, body mass index independently predicted progression of paroxysmal to permanent AF (1.04 per kg/m^2^ (95% confidence interval 1.03 to 1.06)) after adjustment for age and sex.^29^ Thus, compelling evidence links obesity to higher risks of AF across a wide age range and in both sexes. Mendelian randomisation studies use genetic variation to investigate causal relations between modifiable risk factors and health outcomes in observational data; the results of which have supported a causal association between higher body mass index and AF risk.^30^ Mendelian randomisation studies also show that obesity often co-exists with hypertension,^20^ insulin resistance,^31^ and obstructive sleep apnoea,^32^ which each independently confer increased arrhythmic risk (figure 1). In the following sections we explore the extent to which increases in arrhythmic risk in obesity is explained by increases in adipose volume, particularly epicardial fat, or by obesity related comorbidity.

## Epicardial adipose tissue as mediator of atrial fibrillation

Epicardial adipose tissue is the visceral fat depot of the heart and is in intimate contact with the underlying myocardium, covering up to 80% of the epicardial surface. This tissue shares the same embryological origin and circulation as the myocardium, facilitating paracrine cross-talk between these neighbouring tissues.^33^ The epicardial adipose tissue volume increases with increasing obesity,^34^ resulting in fatty myocardial infiltration and relative fat depot hypoxia, causing adipocytes to secrete pro-inflammatory and pro-fibrotic adipocytokines that adversely affect electrophysiological function^35^ (figure 1). Consequently, epicardial adipose tissue volume is positively correlated with P wave duration,^36 37^ amplitude,^36^ and dispersion,^37^ and PR interval prolongation.^38^ Meta-analysis of eight observational studies comprising 375 526 participants reported PR interval prolongation is associated with a 45% increased relative risk of AF (hazard ratio 1.45 (95% confidence interval 1.23 to 1.71)).^39^ Initial evidence that increased epicardial adipose tissue is associated with increased risk of AF was provided by a computed tomography study of 3217 participants in the FHS which showed an AF odds of 28% higher after body mass index and intrathoracic fat deposit adjustment (odds ratio per standard deviation of pericardial fat volume 1.28 (95% confidence interval 1.03 to 1.58)).^40^ Epicardial fat volumes are greater in patients with AF versus people in sinus rhythm and the volume is related to duration and burden of AF, independent of age, sex, left atrial enlargement, hypertension, diabetes, and body mass index.^41 42^ In a meta-analysis of 63 observational studies of 352 275 individuals, every standard deviation increment in epicardial adipose tissue volume was associated with 2.6-fold higher odds of AF, as well as 2.2-fold higher odds of persistent versus paroxysmal AF.^27^ Interestingly, AF risk for every standard deviation increment in anthropometric indices of adiposity was weaker than for epicardial adipose tissue volume (odds ratio of body mass index 1.22 (95% confidence interval 1.17 to 1.27); waist circumference 1.32 (1.25 to 1.41); waist to hip 1.11 (1.08 to 1.14), *v* epicardial adipose tissue 2.61 (1.89 to 3.60)), suggesting arrhythmic risk related to obesity is at least partly attributable to epicardial adipose tissue.

## Mediators of atrial fibrillation risk in obesity

Obesity often co-exists with cardiometabolic diseases, such as hypertension, dyslipidaemia, and insulin resistance, broadly referred to as the metabolic syndrome.^6^ This clustering of cardiometabolic perturbations confers a higher risk of AF.^43^ Obstructive sleep apnoea is also common in obesity and has been linked to AF.^32 44 45^ The extent to which obesity related AF risk is mediated via concurrent hypertension, insulin resistance, and obstructive sleep apnoea will be discussed.

### Hypertension

Obesity causes essential hypertension,^46^ which in turn increases risk of AF. In the Atherosclerosis Risk in Communities cohort study of 14 598 individuals, hypertension was the largest population attributable fraction to AF risk contributing to more than a fifth of incident AF (21.6% (95% confidence interval 16.8 to 26.7)).^20^ Hypertension augments obesity related haemodynamic adaptations, although echocardiographical observations suggest disparate mechanisms of myocardial remodelling between hypertension and obesity.^47^ In a prospective Norwegian population based cohort study with a 35 year follow-up,^48^ incident AF after adjustment for other cardiovascular risk factors increased for people with blood pressures at the upper limit of normal (hazard ratio 1.50 (95% confidence interval 1.10 to 2.03)) and in people in the hypertensive range (1.60 (1.15 to 2.21)). Other evidence suggesting that the higher risks of AF in obesity are mediated by hypertension was provided by the LEGACY cohort study of 355 people, in which weight loss reduced blood pressure and antihypertensive use in tandem with reduction of AF burden.^49^ These studies provide evidence that arrhythmic risks might be partly explained by hypertension when in people with obesity.

### Diabetes

Multisystem lipotoxic effects of increasing adiposity results in insulin resistance and eventually diabetes, which increases AF risk.^50^ In the FHS, diabetes was an independent risk factor for AF (odds ratio of 1.4 for men and 1.6 for women).^31^ A population based case-control study comprising 3613 individuals showed that every percent increment in glycated haemoglobin (HbA1c) was associated with 14% higher adjusted odds of AF increasing by as much as 3% for every year of diabetic treatment.^51^ Similarly in the Atherosclerosis Risk in Communities study, a positive linear association between HbA1c and incident AF risk was noted in people with diabetes.^52^ Additionally, increased HbA1c independently predicts recurrent AF post-ablation,^53 54^ and among 106 patients with a new diagnosis of AF followed up over five years with ambulatory electrocardiogram monitoring, diabetes independently predicted progression from paroxysmal to persistent AF.^55^ A meta-analysis of 25 case-control studies comprising 2932 patients reported reduced heart rate variability in patients with type two diabetes, suggesting autonomic dysfunction.^56^ Diabetic autonomic neuropathy is characterised by parasympathetic denervation resulting in unchecked, increased sympathetic tone that contributes to AF pathogenesis.^57^ A meta-analysis of 8 037 756 individuals reported a higher risk of AF in patients with diabetes even after adjusting for obesity (relative risk 1.22 (95% confidence interval 1.09 to 1.38)).^58^ Diabetes is therefore an arrhythmic risk factor, both independently and in the context of obesity.

### Obstructive sleep apnoea

Obstructive sleep apnoea was associated with AF, independent of body mass index, neck circumference, hypertension, and diabetes, in a prospective cohort study of 463 patients (odds ratio 2.19 (95% confidence interval 1.40 to 3.42)).^32^ An analysis of the Sleep Heart Health Study, a multicentre longitudinal study of 6441 participants that investigated the cardiovascular consequences of sleep-disordered breathing, showed an even greater association with AF after adjustment for age, sex, body mass index, and coronary disease (odds ratio 4.02 (95% confidence interval 1.03 to 15.74)).^44^ A dose dependent relation between obstructive sleep apnoea and AF burden was also reported in an Australian cohort of 6841 patients referred for polysomnography, in whom duration of apnoea and time in hypoxaemia were arrhythmic predictors independent of body mass index.^45^ Similarly, the Outcomes Registry for Better Informed Treatment of Atrial Fibrillation (ORBIT-AF) registry, which enrolled 10 132 participants with AF who were followed up for two years, found that patients with obstructive sleep apnoea had a greater burden of severe or disabling symptomatic AF than did those without (22% *v* 16%, P<0.001), although no differences in risk of AF progression were reported (hazard ratio 1.06 (95% confidence interval 0.89 to 1.28)).^59^

Despite these data, evidence for the link between obstructive sleep apnoea and AF is conflicting. For instance, the Olmsted Country retrospective cohort study of 3542 adults reported that obesity, but not obstructive sleep apnoea, was an independent risk factor for incident AF in adults older than 65 years.^60^ Similarly, the Outcomes of Sleep Disorders in Older Men Study of 2911 men showed that obstructive sleep apnoea severity was associated with risk of cardiovascular events but not with incident AF.^61^ Consistent with these findings, no differences were reported in freedom from arrhythmia after ablation among AF patients receiving standard care or weight and obstructive sleep apnoea management.^62^ Finally, a large retrospective study of 30 188 patients with sleep-disordered breathing (obstructive sleep apnoea or central sleep apnoea) showed that time to recurrent AF after cardioversion or ablation was not influenced by continuous positive airway pressure treatment (hazard ratio 1.01 (95% confidence interval 1.00 to 1.02)).^63^

The conflicting evidence for the effects of obstructive sleep apnoea on AF risk might be partly explained by the confounding effects of concurrent obesity. For instance, although obstructive sleep apnoea is twice as prevalent in patients who are obese compared with people of healthy weight;^64^ this association might be bidirectional and obstructive sleep apnoea can increase susceptibility to weight gain by inducing leptin resistance.^65^ Given these complexities, Mendelian randomisation studies can overcome some limitations of observational data to identify causal links between phenotypes. One Mendelian randomisation study showed that the genetic propensity for obstructive sleep apnoea also increased AF risk (odds ratio 1.21 (95% confidence interval 1.12 to 1.31)).^66^ However, this analysis was based on only five single nucleotide polymorphisms, of which two were also associated with body mass index or fat-free mass at the genome wide significance level, suggesting this link could be mediated by obesity. Indeed, another Mendelian randomisation study including almost sixfold single nucleotide polymorphisms showed that although obstructive sleep apnoea was associated with AF in univariable analysis (odds ratio 2.09 (95% confidence interval 1.10 to 3.98)), this finding became non-significant after adjustment for body mass index (0.68 (0.42 to 1.10)).^30^ In brief, available data suggest that proarrhythmic risks associated with obstructive sleep apnoea are partly mediated by obesity.

## Abnormal ventricular repolarisation and ventricular arrhythmias

The QT interval is a measure of the duration of ventricular activation and repolarisation. Given that action potential duration shortens as heart rate increases, in clinical practice the interval is corrected for heart rate (corrected QT, or QTc). Generally, QTc intervals of 450 ms or more in men and of 470 ms or more in women are considered abnormal. QTc dispersion non-invasively quantifies dispersion of ventricular repolarisation, and is calculated as the difference between the longest and shortest QTc interval on a 12 lead electrocardiogram. This term refers to spatial heterogeneity of repolarisation, such that increased dispersion can result in re-entry thereby facilitating ventricular tachycardia or fibrillation. Although the clinical usefulness of QTc dispersion over QTc interval in predicting adverse arrhythmic and cardiovascular outcomes has been debated,^67^ both prolong with obesity and reverse with weight reduction.^68–72^ A comprehensive meta-analysis of the effects of the obesity on ventricular repolarisation showed that QTc was longer in individuals who were obese versus people who were not (mean difference 21.74 ms (95% confidence interval 18.76 to 32.32)).^68^ Similarly, pooled estimates for QTc dispersion were greater in obesity (15.17 ms (13.59 to 16.74)). Another meta-analysis of 22 studies showed that QTc shortened and QTc dispersion decreased after weight loss induced by lifestyle or surgery (QTc, mean difference −25.77 ms (95% confidence interval −18.86 to −28.21); QTc dispersion, −13.46 ms (−11.32 to −15.60)).^69 71^

Consistent with abnormal ventricular repolarisation associated with obesity, increasing body mass index has also been associated with higher risk of ventricular arrhythmias and sudden cardiac death^73^ (figure 1). However, this risk might not be attributable to only obesity given the variable definitions of sudden cardiac death that could also include fatal cardiovascular events resulting from comorbidity. Nevertheless, one meta-analysis of 14 prospective studies reported relative risks for sudden cardiac death of 1.16 (95% confidence interval 1.05 to 1.28) per 5 unit increment in body mass index, 1.82 (1.61 to 2.07) per 0.1 unit increase in waist to hip ratio, and 1.03 (0.93 to 1.15) per 10 cm increase in waist circumference.^73^ In the MADIT trial, individuals who were obese received a higher rate of appropriate shock treatment from implantable cardiac defibrillators than individuals who were not obese (39% *v* 24%, p=0.014).^74^ The higher burden of ventricular ectopy, particularly in the context of left ventricular hypertrophy,^75^ might partly explain the higher incidence of ventricular arrhythmias and sudden cardiac death in obesity, whereby critically timed extrasystoles can trigger re-entrant arrhythmias in a remodelled substrate.^76^ The risks of ventricular arrhythmias and sudden cardiac death in obesity are likely to be mediated by increasing adiposity as well as cardiometabolic comorbidity that independently cause electrophysiological remodelling.

## Weight loss strategies to mitigate obesity related risk of atrial fibrillation

**Figure 2:**
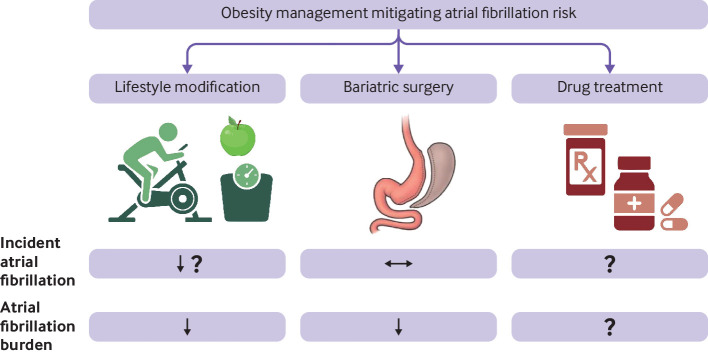
Management options for obesity and their effects on atrial fibrillation risks. Although lifestyle changes and bariatric surgery reduces atrial fibrillation burden in obese patients with pre-existing atrial fibrillation, the evidence for surgical weight reduction on mitigating the risk of incident atrial fibrillation is conflicting. Lifestyle changes are likely to reduce incident atrial fibrillation risk. The effects of pharmacological modulation of obesity on incident atrial fibrillation risk and atrial fibrillation burden is not well described

The anti-arrhythmic effects of weight loss treatments have gained much interest. Here, we review weight management strategies for reducing AF risk and burden and are summarised in figure 2.

### Non-surgical weight reduction interventions

Given that every 5 unit increment in body mass index confers about 30% greater risk of incident AF, 13% increase in recurrent AF post-ablation, and 10% increase in post-surgical AF,^26^ weight loss was hypothesised to potentially alleviate AF burden. This theory was tested in a single centre, partially blinded study, in which 150 ambulatory patients who were overweight or obese with symptomatic paroxysmal AF were randomly assigned to an active weight management programme or offered general lifestyle advice.^77^ Participants randomly assigned to active weight management experienced on average 14.3 kg weight reduction compared with 3.6 kg in the control group, as well as improvements in AF symptom burden scores (11.8 *v* 2.6 points) and symptom severity scores (8.4 *v* 1.7 points). This occurred in the context of reduced number of AF episodes, cumulative AF duration, interventricular septal thickness, and left atrial area in those randomised to active weight management, suggesting reverse remodelling of atrial tissue with weight loss.

The ARREST-AF cohort study offered 281 patients with a body mass index of 27 or more and hypertension, glucose intolerance or diabetes, hyperlipidaemia, obstructive sleep apnoea, smoking, or alcohol excess, undergoing catheter ablation for symptomatic AF either aggressive risk factor management or standard care.^78^ Risk factor management resulted in significantly greater blood pressure reductions (34.1 mm Hg (standard deviation 7.5) *v* 20.6 mm Hg (standard deviation 3.2), P=0.003), and better glycaemic control (HbA1c concentrations of <7% in 100% *v* 29%, P=0.001) and lipid profiles (46% *v* 17%, P=0.01) compared with the control group. AF-free survival was greater in the risk factor management arm, and furthermore, risk factor management independently predicted AF-free survival after multiple ablations. In the context of comprehensive blood pressure and glycaemic, lipid, and weight management, the extent to which weight loss contributed to the reduction in AF burden is not possible to ascertain.

In the LEGACY cohort study, 355 patients who were obese with AF receiving risk factor management were followed up over a mean of four years.^49^ The degree of weight loss related to freedom from AF and individuals maintaining more than 10% weight loss had a sixfold greater probability of arrhythmia-free survival compared with patients with 3-9% or less than 3% weight loss. Importantly, the number of anti-arrhythmic drugs or ablation procedures were similar between groups at baseline, and degree of weight loss corresponded with participation in weight management clinic suggesting a positive reinforcement effect. AF-free survival associated not only with weight loss but also subsequent weight maintenance, such that AF burden and symptom severity was less in those with less than 2% weight fluctuation compared with those with more than 5%.^49^

The implications for weight loss on AF in the LEGACY cohort was further characterised in the REVERSE-AF study comprising 355 individuals.^79^ Approximately 48 (40%) of 116 individuals with less than 3% weight loss progressed from paroxysmal to persistent AF and a quarter reversed from persistent to paroxysmal or no AF. Of the individuals who had 3-9% weight loss, a third (n=33/104) progressed from paroxysmal to persistent AF and almost half reversed from persistent to paroxysmal or no AF. Among individuals recording more than 10% weight loss, only 3% progressed to persistent AF, and almost 90% reversed from persistent to paroxysmal or no AF. Taken together, the degree of weight loss and its maintenance are likely important lifestyle changes assisting in alleviating arrhythmic burden. Importantly, these results show that early weight management intervention helps to mitigate and even reverse progression of AF to prevent irreversible atrial remodelling. The dose response effect of weight loss and improved fitness on AF burden was corroborated in a retrospective cohort study of 69 885 patients undergoing treadmill stress tests which showed that every one metabolic equivalent increment during exercise was associated with 7% lower risk of incident AF (hazard ratio, 0.93; 95% confidence interval 0.92-0.94; P<0.001), and that this effect was more pronounced in obese individuals and independent of baseline fitness.^80^ In short, there is compelling evidence supporting weight reduction to alleviate arrhythmic burden.

Orlistat, a lipase inhibitor, is the only guideline recommended drug for obesity management in the UK,^81^ however its effects on arrhythmia reduction are not well described. Recently, glucagon-like peptide-1 receptor agonists (GLP-1RA) have emerged as novel drugs in obesity, irrespective of diabetic status.^82–89^ The first to be approved by the Food and Drug Administration and European Medicine Agency was liraglutide, followed by semaglutide by the FDA in June 2021. Although GLP-1RAs appear to have bridged the weight reduction gap between lifestyle and surgical weight reduction strategies, which typically result in less than 10% and more than 20% weight loss respectively,^90^ their anti-arrhythmic effects when prescribed for weight reduction remain unproven. In the context of treating diabetes, a meta-analysis of 31 randomised trials comprising about 18 000 patients with diabetes on GLP-1RA versus 15 000 controls concluded that GLP-1RAs did not influence AF incidence.^91^

### Bariatric surgery

Bariatric surgery is a cost effective intervention for moderately to severely obese people compared with non-surgical interventions.^92^ Despite compelling evidence suggesting weight loss reduces arrhythmic burden in those with pre-existing atrial arrhythmias,^93^ conflicting evidence exists for the effect of bariatric surgery on incident AF risk.^94 95^ One meta-analysis comprising 39 prospective and retrospective cohort studies showed that bariatric surgery reduced all-cause mortality (pooled hazard ratio 0.55 (95% confidence interval 0.49 to 0.62)), cardiovascular mortality (hazard ratio 0.59 (95% confidence interval 0.47 to 0.73)), and incidence of heart failure (0.50 (0.38 to 0.66)), myocardial infarction (0.58 (0.43 to 0.76)), and stroke (0.64 (0.53 to 0.77)).^96^ However, no statistical reduction was noted in incident AF (hazard ratio 0.82 (95% confidence interval 0.64 to 1.06)).^96^ Of the 39 studies included in this meta-analysis,^96^ only seven listed AF in their primary or secondary outcomes, of which only three studies assessed incident AF as a primary outcome measure after bariatric surgery. Consequently, the absence of any effect of bariatric surgery on incident AF risk might be partly explained by the relative paucity of studies investigating AF as their primary objective.

A reduction in AF after bariatric surgery has also been reported in the context of a reduction in obesity related comorbidities. For example, in a longitudinal study of 827 patients who were morbidly obese and who underwent laparoscopic adjustable gastric banding, incident AF was significantly greater in patients with obstructive sleep apnoea than in those without obstructive sleep apnoea (1.7% *v* 0.5%, P<0.001).^97^ By contrast, among the 5067 participants in the Look AHEAD (Action for HEAlth in Diabetes) cohort, weight loss from lifestyle modification in overweight and obese individuals with type 2 diabetes did not reduce incident AF risk.^98^ The absence of the preventive anti-arrhythmic effect might be due to the relatively modest body weight reductions achieved by lifestyle measures compared with bariatric surgery (about 10% *v* 20%) and is consistent with observations from studies supporting sustained weight reduction for greater arrhythmia-free survival and improvements in cardiovascular outcomes.^49 77 99^ Similarly, among a national primary and secondary healthcare database of more than 11 million patients in the UK, although rate of AF resolution among patients undergoing bariatric surgery was greater than among matched controls for age, sex, and body mass index, a downward but non-significant trend for AF incidence was reported (adjusted hazard ratio 0.72 (95% confidence interval 0.49 to 1.05)).^95^ In summary, although surgical and non-surgical weight reduction is an effective intervention for reducing AF recurrence, the evidence for bariatric surgery reducing incident arrhythmic risk is less consistent.^71^

## Evidence for reduction in sudden cardiac death risk with weight loss

Although obesity has been associated with a higher risk of sudden cardiac death in large multicentre, prospective cohort studies,^73 100–102^ no studies to date have quantified any reduction in risk following weight loss. This lack of data is perhaps unsurprising given the practical challenges in anticipating terminal events and contribution of morbidity. However, given that obesity is also associated with a higher risk of ventricular tachycardia or fibrillation^74 103^ and weight loss with electrocardiogram changes suggestive of favourable changes in ventricular activation and repolarisation,^68 69 104 105^ it may be inferred the weight loss at least partly mitigates sudden cardiac death risk in obesity.

## Anti-arrhythmic effects of treatments for obesity related comorbidities

**Figure 3:**
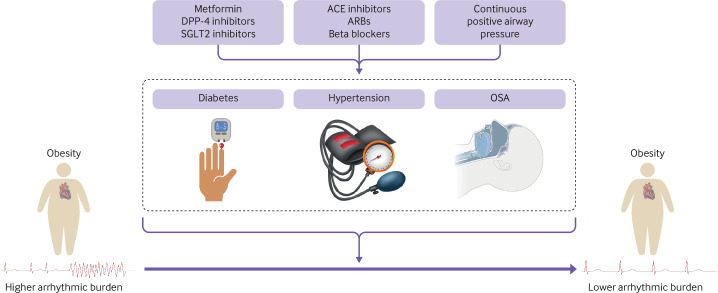
Management of diabetes, hypertension, and obstructive sleep apnoea (OSA) have additional anti-arrhythmic effects. Arrhythmic risk in obesity is partly mediated by concurrent cardiometabolic disease and obesity related ill health, such as OSA. Their respective treatments might alleviate arrhythmic burden in the long term. DPP-4=dipeptidyl-peptidase-4 inhibitors; SGLT2=sodium-glucose cotransporter-2; ACE=angiotensin-converting enzyme; ARBs=angiotensin receptor blockers

A detailed review of evidence based management for comorbidities related to obesity is beyond the scope of this review. Instead, we specifically outline the evidence for the treatment of hypertension, obstructive sleep apnoea, and diabetes in patients who are obese that might confer anti-arrhythmic benefits, in addition to weight reduction highlighted in the previous section (figure 3).

### Anti-hypertensive treatments

Obesity and chronic hypertension independently cause left ventricular hypertrophy, which has been associated with a higher risk of atrial and ventricular arrhythmias.^106^ In the double blind, placebo controlled, TRACE study comprising 790 patients randomly assigned to trandolapril treatment and 787 to placebo, the incidence of AF over a four year follow-up was greater in the trandolapril group compared with the placebo group (5.3% *v* 2.8%, P<0.05). Adjusted multivariable regression analysis suggested trandolapril treatment independently reduced the risk of new AF (risk ratio 0.45 (95% confidence interval 0.26 to 0.76)) in people with left ventricular dysfunction after a myocardial infarction.^107^ Similarly, angiotensin receptor blockers in combination with anti-arrhythmic treatment have been shown to reduce AF burden in patients with persistent AF, defined as an episode of AF lasting for more than seven days.^108^ Despite the short two month follow-up duration, the intention-to-treat analysis of 154 patients in a randomised trial showed that people prescribed irbesartan in addition to amiodarone had lower rate of recurrent AF (84.79% *v* 63.16%) as well as a greater probability of remaining AF-free (79.52% *v* 55.91%).^108^ First line agents for hypertension treatment can therefore confer additional anti-arrhythmic effects.

### Continuous positive airway pressure

Continuous positive airway pressure (CPAP) is the mainstay treatment for obstructive sleep apnoea,^109^ although reports on the effects of this treatment on AF risk are conflicting. Observational studies have reported reductions in atrial and ventricular ectopy^110^ and arrhythmias^111^ with continuous positive airway pressure and maintenance of sinus rhythm after cardioversion and catheter ablation for AF.^112^ Two meta-analyses of 108 observational studies evaluating the effect of continuous positive airway pressure on AF risk reported similar results. One meta-analysis of 3743 people suggested that AF risk increased by almost 60% in people with obstructive sleep apnoea who were not treated with continuous positive airway pressure.^113^ The other meta-analysis of 4752 patients reported a 42% relative risk reduction of AF recurrence with continuous positive airway pressure among more than 1000 patients.^114^ Another meta-analysis of clinical and observational studies comprising 14 812 patients showed that continuous positive airway pressure reduced AF recurrence by over 60%.^115^ The benefits of this treatment in reducing AF recurrence might also be greater in younger patients (<60 years).^116^ Similarly, continuous positive airway pressure has been shown to be particularly efficacious at decreasing AF risk in younger men who are obese, supporting intervention early in the progression of obesity.^117^

Nevertheless, an open label randomised trial of 108 patients with paroxysmal AF and moderate to severe obstructive sleep apnoea, showed no difference in AF burden between groups treated with continuous positive airway pressure and the standard care group.^118^ Likewise, in a randomised trial of 37 patients receiving continuous positive airway pressure and 46 patients receiving standard care, no difference was reported in AF burden after pulmonary vein isolation between groups.^119^ Discrepancies between observational and randomised data might be due to selection of individuals with minimal symptoms in clinical trials. Patients with symptoms are more likely to be established on CPAP and physicians might be reluctant to recruit them to studies where the patients are randomly assigned to a control arm. Furthermore, incomplete CPAP adherence in patients who have minimal symptoms can confound results. Nevertheless, a Mendelian randomisation study of data from 55 114 patients with AF and 482 295 controls, published in 2022, showed that sleep disordered breathing was not associated with AF after adjusting for body mass index.^30^ Importantly, however, body mass index remained associated with AF after adjusting for obstructive sleep apnoea. This finding suggests that the link between obstructive sleep apnoea and AF might be mediated or confounded by concurrent obesity in patients with mild-to-moderate obstructive sleep apnoea^30^ and supports aggressive strategies for weight reduction to mitigate AF risk in these patients.

### Oral hypoglycaemic agents

Metformin is the first line agent for type two diabetes, with sulphonylureas, thiazolidinediones, dipeptidyl-peptidase 4 inhibitors, GLP-1RAs, and sodium-glucose cotransporter-2 inhibitors (SGLT2i) considered second line or third-line options.^120^ Insulin is useful when other drugs do not result in normoglycaemia, or in patients with type one diabetes, in which insulin is a first-line treatment. The absence of arrhythmic endpoints in prespecified primary outcomes is a limitation of many trials. For this reason, the evidence for any anti-arrhythmic properties of drugs intended for other clinical indications is often limited to retrospective post-hoc analysis. Nevertheless, metformin has been associated with a decreased risk of AF^121^ and AF recurrence after catheter ablation.^122^ SGLT2i have been shown to confer beneficial cardiovascular effects, particularly in reducing major adverse cardiovascular events.^123–127^ Consistent with improvements in rates of myocardial infarction and heart failure events, meta-analyses of randomised controlled trials have shown reductions of atrial and ventricular arrhythmias with SGLT2i treatment^128 129^ that are greater than those observed with dipeptidyl-peptidase-4 inhibitors.^130^ One meta-analysis of 31 randomised studies (n=75 279) showed that SGLT2i reduced AF related events compared with placebo or no treatment (1.1% *v* 1.5%; risk ratio 0.75 (95% confidence interval 0.66 to 0.86)).^129^ Another analysis of 34 randomised trials consisting of more than 63 000 patients aged 53-67 years followed up between 24 weeks and 5.7 years, SLGT2i treatment was associated with lower incidence of atrial arrhythmias (odds ratio 0.81 (95% confidence interval 0.69 to 0.95)), but not of ventricular arrhythmias or cardiac arrest.^128^ Notably, not all studies in this meta-analysis reported arrhythmic events, and even in those that did, the overall incidence was 1% for atrial arrhythmias and less than 0.5% for ventricular arrhythmias and sudden cardiac death. Nevertheless, a post-hoc analysis of the Dapagliflozin and Prevention of Adverse Outcomes in Heart Failure trial (DAPA-HF) showed reduced a risk reduction of 21% in ventricular arrhythmias and cardiac arrest in the dapagliflozin arm (hazard ratio 0.79 (95% confidence interval 0.63 to 0.99), although ventricular arrhythmias may have been underreported as they did not constitute a prespecified outcome.^131^ Similarly, in another post hoc analysis, dapagliflozin was associated with a 19% relative risk reduction in incident atrial arrhythmias compared with placebo among more than 17 000 patients who are diabetic, independent of any arrhythmic history.^132^

## Emerging treatments

The EMPA-ICD study (jRCTs031180120) is underway and will examine SGLT2i effects on ventricular arrhythmias in patients with diabetes and an implanted defibrillator, clarifying whether their indications will extend beyond current guidelines. Similarly, ERASE, a multicentre, phase 3 study (NCT04600921), will report on the effects of ertugliflozin on ventricular arrhythmias in patients with heart failure irrespective of diabetic status. For AF burden, EMPA-AF (NCT04583813) will also report on the effect of empagliflozin and DAPA-AF (NCT04792190) will investigate use of dapagliflozin.

## Guidelines

Guidelines for arrhythmia management include reference to weight management as a modifiable risk factor. The 2020 European Society for Cardiology guidelines for the diagnosis and management of AF developed in collaboration with the European Association for Cardio-Thoracic Surgery state that "given the potential to reduce AF episodes by weight reduction, AF catheter ablation should be offered to obese patients in conjunction with lifestyle modifications for weight reduction."^133^ Furthermore, patients who are overweight or obese that require radiofrequency ablation for AF should aim for more than a 10% weight reduction and a target body mass index of less than 27 to optimise outcomes.^133^ The guidelines mention that "weight loss, strict control of risk factors, and avoidance of triggers for AF are important strategies to improve outcome of rhythm control."^133^ The 2019 American Heart Association, American College of Cardiology, and Heart Rhythm Society also recommend weight loss for patients with AF who are overweight and obese.^134^

## Conclusion

Obesity is a complex multisystem disorder that causes compensatory cardiovascular adaptations, culminating in electromechanical dysfunction. Atrial and ventricular electrophysiological remodelling in obesity manifest as detectable electrocardiographical changes. In atrial tissue, these changes can be explained by increased fibrosis, reduced conduction velocity, and increased conduction heterogeneity. Increasing adiposity, especially epicardial fat, is detrimental to atrial and ventricular electrophysiology due to paracrine effects and fatty infiltration, promoting a proarrhythmic substrate. Furthermore, the haemodynamic, lipotoxic, and mechanical consequences of increasing body mass results in cardiometabolic and ventilatory perturbations, such as hypertension, diabetes, and obstructive sleep apnoea. AF risk that is related to obesity is likely to be also partly mediated by these phenotypes. Compelling evidence suggests that lifestyle and surgical weight loss reduces AF burden, but evidence for a reduction in incident AF risk by either intervention remains uncertain. Evidence shows that treating hypertension, diabetes, and obstructive sleep apnoea might have additional anti-arrhythmic effects. Most recently, novel drugs have been identified to induce weight loss to a degree that is similar to bariatric surgery, although effects on reducing arrhythmias is yet to be established.

**Questions for future research**

- To what extent can any subclinical electrophysiological remodelling in arrhythmia-free morbidly obese (body mass index >40) patients be reversed with lifestyle or surgical weight reduction interventions?
- To what extent do primary prevention weight reduction strategies reduce incidence of atrial and ventricular arrhythmias in morbidly obese (body mass index >40) patients?
- How can obesity management be effectively delivered to groups who are at risk and in whom weight loss might be difficult to maintain, such as patients with mobility problems or learning difficulties?
- What are the long term outcomes of bariatric surgery in children and young people with obesity?

**Patient involvement**

No patients were asked for input in the creation of this article.
